# Bilateral Chondroepitrochlearis Muscle: Case Report, Phylogenetic Analysis, and Clinical Significance

**DOI:** 10.1155/2016/5402081

**Published:** 2016-05-08

**Authors:** Sujeewa P. W. Palagama, Raymond A. Tedman, Matthew J. Barton, Mark R. Forwood

**Affiliations:** ^1^School of Medicine, Griffith University, Gold Coast, QLD, Australia; ^2^Post Graduate Institute of Medicine, University of Colombo, Colombo, Sri Lanka; ^3^Menzies Health Institute Queensland, Griffith University, Gold Coast, QLD, Australia; ^4^School of Medical Sciences, Griffith University, Gold Coast, QLD, Australia

## Abstract

Anomalous muscular variants of pectoralis major have been reported on several occasions in the medical literature. Among them,* chondroepitrochlearis* is one of the rarest. Therefore, this study aims to provide a comprehensive description of its anatomy and subsequent clinical significance, along with its phylogenetic importance in pectoral muscle evolution with regard to primate posture. The authors suggest a more appropriate name to better reflect its proximal attachment to the costochondral junction and distal attachment to the epicondyle of humerus, as “chondroepicondylaris”; in addition, we suggest a new theory of phylogenetic significance to explain the twisting of pectoralis major tendon in primates that may have occurred with their adoption to bipedalism and arboreal lifestyle. Finally, the clinical significance of this aberrant muscle is elaborated as a cause of potential neurovascular entrapment and as a possible hurdle during axillary surgeries (i.e., mastectomy).

## 1. Introduction

There are many anatomical variations in human musculature. Some variations are common while others have been infrequently reported. When present, anatomical variations of pectoralis major (PM) muscle are noteworthy, not only because of their extreme rarity but also for their phylogenetic and clinical significance. Many different anomalies associated with PM have been extensively described by Bergman and colleagues, such as* chondroepitrochlearis muscle* (CEM);* chondrofascialis*;* pectoralis quartus*;* sternalis;* and* axillary arch muscle* (Achselbogen of Langer) [1] out of which the latter is the most frequently reported anomaly [2].

**Figure 1:**
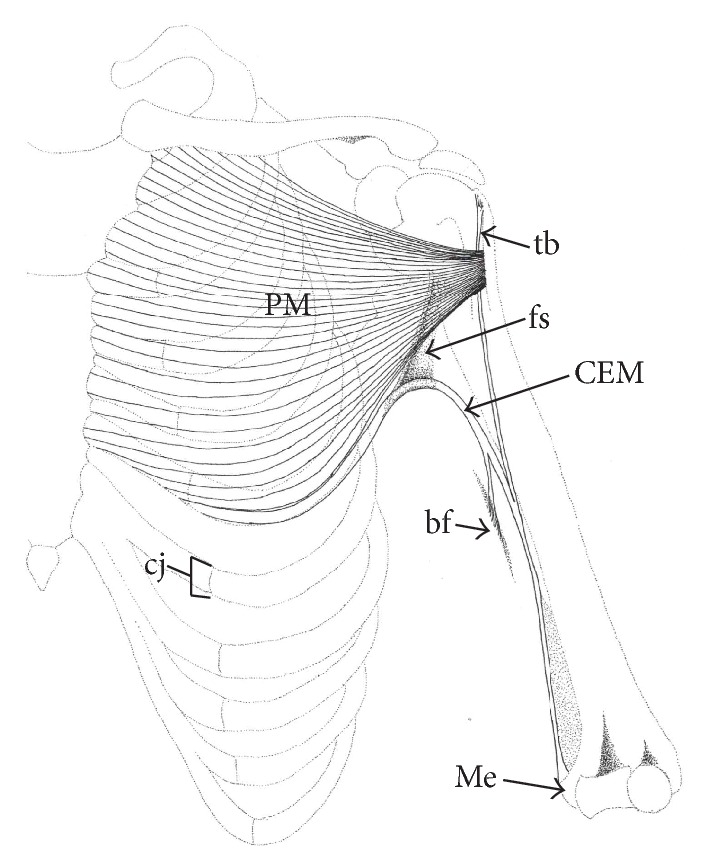
Schematic drawing of the left thorax and upper limb, demonstrating the chondroepitrochlearis muscle (CEM) inserting into the deep brachial fascia (bf) and the fibrous band (tuberoepicondylar band, tb) (PM: pectoralis major; fs: fascial sling; cj: costochondral junction; and Me: medial epicondyle).

CEM is an exceptionally rare anomaly associated with PM [3]. CEM is a muscle that originates from the cartilages or costochondral junctions of the thorax and inserts into the distal brachium (Figure 1); consequently, the most common name that has been used for this variation is chondroepitrochlearis. After it was first described by Duvernoy (who named it) in 1855, as cited by Wood [4], only a few specimens of this clinically important variant have been reported in medical literature. A research group demonstrated just one CEM specimen out of 200 cadavers during their 20 years of study [5]. Meanwhile, despite a vigilant detailed exploration to identify this particular PM variant, another group was unable to demonstrate its presence even after 107 cadavers dissections [6], signifying its rarity. Even among these rare occasions CEM has, on record, most been associated with other muscular anomalies such as* axillary arch muscle* and* pectoralis quartus* [7], with the former being the most common coassociation [3, 8–12]. Therefore, the presence of CEM with no other muscular anomaly should be considered particularly rare [13, 14]. Moreover, this reported case is the second of its kind with a bilateral presence that is devoid of other muscular anomalies, preceded only by [5]. However, this specimen has many distinct and unique features, which are yet to be described in the known medical literature.

The purpose of this study was to present a complete account of the gross anatomy of this rare anomaly and to explain its features in terms of phylogenetic significance and the relevance it may have to contemporary clinical practice.

## 2. Case Report

**Figure 2:**
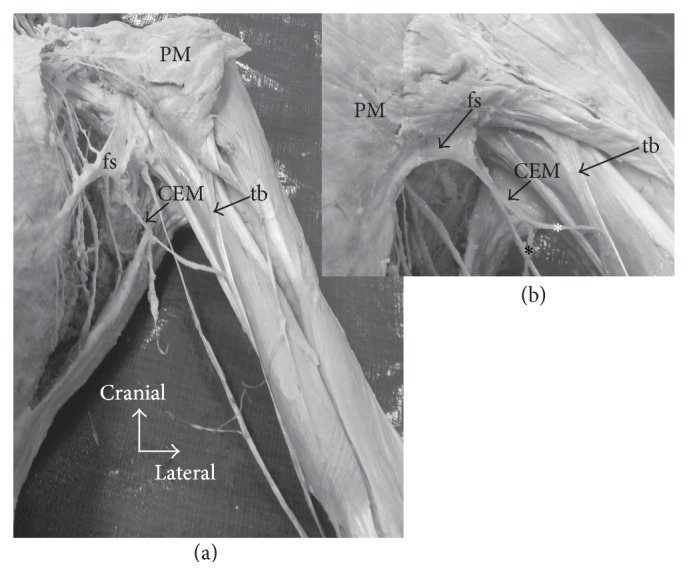
Photographs of the left chondroepitrochlearis muscle (CEM): (a) highlights the course of the CEM from its origin in the thorax through to its insertion into the fibrous band (tuberoepicondylar band, tb) (note: pectoralis major (PM) has been reflected cranially). (b) insert from (a) that highlights the course of the CEM in the axilla and its suspension by the fascial sling (fs). The photograph depicts two separate CEM slips at insertion. One slip inserts into the deep brachial fascia (black asterisk) and the other (white asterisk) fuses with the tb.

**Figure 3:**
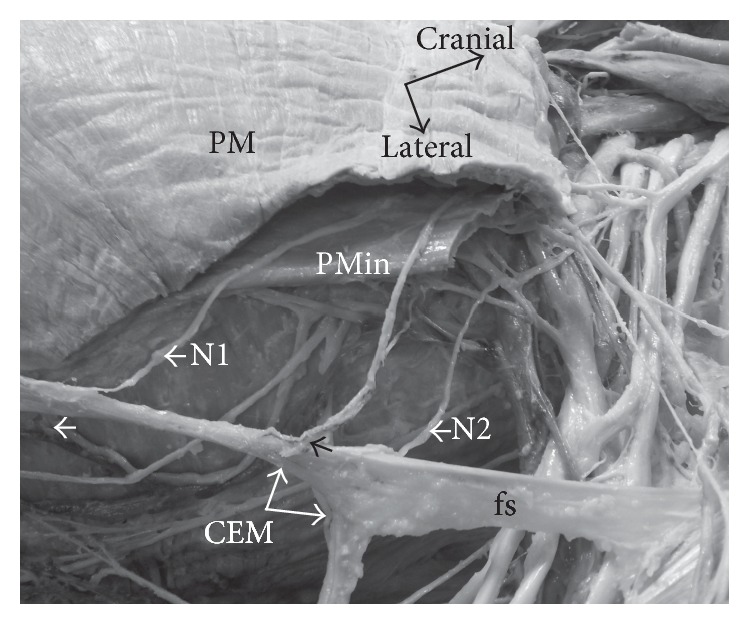
Photograph demonstrating the neurovascular supply of the left CEM. One branch of medial pectoral nerve (N1) passes though pectoralis minor (PMin) and the other branch (N2) passes deep to PMin. Pectoral branch of thoracoacromial trunk (black arrow) provides its arterial supply and lateral thoracic vein (white arrow) drains its venous blood (PM: pectoralis major; fs: fascial sling).

All material was made available by the School of Anatomy, Griffith University, in accordance with the Queensland Transplantation and Anatomy Act, 1979, and the signed informed consent of the donor. The chondroepitrochlearis muscle (CEM) was detected during a routine dissection on one formalin fixed female cadaver (71 years of age). Once the anomaly on the right side of the cadaver was identified and dissected, the left side was dissected in the same method. The CEM on each side was identified as a separate thin muscular slip, adjacent to the inferolateral border of pectoralis major (PM) measuring 22 cm (muscle belly) in length and 0.7 cm in width at its broadest point (Figure 2). Commencing at the costochondral junction of the 5th rib, fibers of CEM took an initial course parallel to yet independent of the lateral margin of PM. After leaving the PM at the axilla, CEM curved inferolaterally, approximately 1.5 cm below the anterior axillary fold. At the axilla, the muscle belly of CEM was suspended to the axillary fascia by a fascial sling (Figures 2 and 3). At the medial border of the brachium, the muscle belly of CEM bifurcated into two separate slips (Figure 2): one is continuous with the deep brachial fascia (Figure 1) while the other inserted into a distinct fibrous band 16 cm above the medial epicondyle of the humerus (Figures 1 and 2).

The anomalous CEM fibrous band originated from the greater tubercle of humerus, deep to the tendinous insertion of PM, and took an oblique course superficial to biceps brachii (BB), median nerve (MN), brachial artery, and basilic vein. The band traversed deep to the medial cutaneous nerve of forearm, where it met a slip of CEM at the medial intermuscular septum, and continued distally to insert onto the medial epicondyle of humerus. The band did not cross the ulnar nerve nor did it have any fibrous connections to it. The ulnar nerve ran posteriorly and parallel to the tendon. This long and slender fibrous band measured 29 cm in total length and 0.5 cm in width at its broadest point. CEM and its tendon were identical in all anatomical characteristics bilaterally.

The nerve supply for CEM was by two small nerves that entered its muscle belly at the lateral border of PM (Figure 3). One nerve could be traced back through pectoralis minor (PMin), while the other nerve ran deep to PMin; however both nerves branched out of brachial plexus from the anterior division of middle trunk (Figure 3). The arterial supply for CEM was from the pectoral branch of thoracoacromial trunk and from the lateral thoracic artery via its fine branches that entered the muscle belly at the lateral chest wall (Figure 3). Venous drainage was traced to the lateral thoracic vein (Figure 3). The neurovascular supply to CEM was identical bilaterally.

There were two other noteworthy anomalies of PM itself and of the brachial plexus. The typical twist of the tendinous insertion of PM was absent bilaterally, while the clavicular fibers inserted most proximally onto the lateral lip of the bicipital groove. The manubriosternal fibers from the 2nd, 3rd, 4th, and 5th ribs were attached distally, relative to the insertion of the clavicular fibers, while the costal fibers from ribs 6, 7, and 8 demonstrated a partial twist and were attached posteriorly to the manubriosternal fibers but more distally, instead of their typically proximal attachments [15]. There was no* axillary arch of Langer* or any other muscle anomalies on the cadaver. In the brachial plexus, there was an aberrant “ansa pectoralis” connecting the anterior divisions of the superior and middle trunks. The lateral pectoral nerve exited from this ansa pectoralis, while medial pectoral nerves branched directly from the anterior division of middle trunk (Figure 3).

## 3. Discussion

### 3.1. Nomenclature

The naming of aberrant muscles has always been a subject for continual amendments in the history of anatomy. Most of the anomalies associated with pectoralis major (PM) have been named as* chondroepitrochlearis muscle* (CEM),* costoepitrochlearis*,* chondrohumeralis,* and* chondrofascialis* characterizing their origin and insertions [1, 21]. As a muscle that arose from the cartilages or costochondral junctions, with an insertion into the distal humerus, the most common name for this PM variation has traditionally been the CEM (Table 1) which was first named by Duvernoy as cited by Wood in 1966 [4]. However, Landry Jr. opposed Duvernoy's claim and questioned whether he had actually seen this muscle in a human cadaver [15]. Nonetheless, as the name suggests, CEM should originate (proximal attachment) from the costal cartilage or costochondral junction and insert onto an area above the trochlear of the humerus. However, the vast majority of documented CEM specimens in the medical literature have inserted onto the medial epicondyle of the humerus (Table 1), questioning the accuracy of the latter half of its name [5, 16, 19].

In order to rectify this inconsistency, a newer nomenclature was proposed by Loukas and colleagues as “thoracoepicondylaris” reflecting a more accurate insertion (distal attachment) point at the humeral epicondyle [19]. Yet, in this new name, the proximal attachment was denoted as “thoraco” which is somewhat vague and suggests that the muscle may arise from anywhere upon the thorax. Such imprecision is inadequate for standard anatomical nomenclature, especially when consistent attachment points for CEM have been frequently described (Table 1). According to a review of literature (Table 1), almost all the specimens of CEM described to date, including the present specimen, have arisen from either a cartilage or a costochondral junction or from the lower fibers of PM which themselves arose from the costal cartilages of the lower ribs [22]. Therefore, “chondro” as the first part of the muscle's nomenclature, first coined by Duvernoy in 1855 [4], appears most appropriate. We suggest that combining this first half with the latter half suggested by Loukas to be “chondroepicondylaris” would better reflect its anatomical features more precisely.

### 3.2. Unique Anatomy of CEM

The basic anatomy of the present case parallels that by Perrin's depiction of the “compound variety” of CEM [16]. This specimen consists of similar basic pattern, morphology, and course to that described by Perrin; however, it varies in its proximal and distal attachment points and its association with an anomalous fibrous band. The CEM in the present study originates from the 5th costochondral junction and consists of two slips at its distal attachments with one inserting into the deep fascia of the brachium, while the other is inserting onto an anomalous fibrous band (Figure 1).

The directional arrangement of the CEM's belly is distinct from other specimens in the literature. It demonstrates a concave shape within the axilla, which is supported by a simple fatty fascial sling, tethering its belly to the axillary fascia. Perrin reported that the CEM formed the anterior axillary fold thus being closely related to the lower border of PM in the axilla [16]. Conversely, however, other authors described CEM to be separated from the anterior axillary fold through a number of mechanisms. A case in point is that Landry described this muscle to be anchored by a tendon that descended from the shoulder capsule [15]; Sarikcioglu et al. described separation by an aponeurosis [14]; Flaherty and associates found it suspended by a neurovascular bundle [5] while Spinner et al. found it in continuity with the brachial plexus in the axilla [11], whereas Chiba and colleagues demonstrated its connection to the axillary arch muscle [3]. However, the current specimen displayed no supporting connection to neurovascular structures within the axilla; rather it was maintained by a simple fascial sling (Figure 3).

#### 3.2.1. An Abnormal Fibrous Band

Unlike other muscles, where their tendons form a continuation of its own belly, the CEM inserted onto an anomalous band which itself had a separate origin (proximal attachment). This band originated from the proximal end of the humerus, while the CEM's belly fused at the middle of its length (12.5 cm below its origin at the greater tubercle) along the medial border of the arm (Figures 1 and 2). A number of authors described a similar band or tendon, yet with a distinct origin [3, 5, 12, 16], although Bryce described a fleshy CEM belly inserted ~2 inches above the medial epicondyle, which lacked any description of a separate tendon [17]. Perrin described that this tendon arose from the joint capsule [16], while Chiba and Ohtani claimed an aponeurotic origin from the tendinous insertion of PM [3, 12]. In the present case, the band of CEM originated from the greater tubercle of the humerus and took an oblique course, crossing the biceps to join the intermuscular septum distally. This accentuated obliquity can be explained by the relative lateral origin being at the greater tubercle (Figure 1). Considering that this fibrous band runs an independent course of that of the CEM and has yet to be named in the literature, we propose the “tuberoepicondylar band” (tb) for nomenclature purposes.

This specimen demonstrated a twofold insertion (Figure 1) of CEM, similarly to other reports which described one insertion into the medial epicondyle (Me) through a unique tendon and the other insertion into the deep brachial fascia (bf, Table 1) [17, 20]. The insertion into the deep brachial fascia has previously been described as the* chondrofascialis* [21, 23]. Since all CEM specimens described in the literature (Table 1) have a common distal attachment to the medial epicondyle, it can be inferred that this carries a phylogenetic significance, which will be discussed later.

#### 3.2.2. Neurovascular Supply

Among the few descriptions of CEM in the literature, only a few have described the innervation of this muscle. Nevertheless, it has been demonstrated that CEM is innervated by the medial pectoral nerve (MPN) [19–21]; however Bryce traced innervations back to the anterior divisions of the brachial plexus [17], while Chiba et al. described two nerve branches arising from the most caudal branch of* ansa pectoralis*, both beneath pectoralis minor (PMin) [3]. Spinner and Flaherty, like Chiba et al., depicted a dual innervation from the confluence of the medial and lateral roots of the median nerve and as a branch of MPN [5, 11]. The present CEM specimen was also dually innervated; firstly, one nerve was traced through the belly of PMin (Figure 3) while the other ran deep to it; however, both did originate from the medial root of* ansa pectoralis* (Figure 3). It was concluded that these two branches were from the medial pectoral nerve as they both innervated PMin as well.

The arterial supply of CEM has been described as being from a branch of thoracoacromial trunk [19], while Barcia, Samuel, and Sarikcioglu demonstrated that it was supplied by branches of the lateral thoracic artery [14, 20, 21]. The present specimen demonstrated combinations of both, as small arteries arose from both the pectoral branch of thoracoacromial trunk and lateral thoracic artery (Figure 3). The venous drainage was through to a tributary of the lateral thoracic vein (Figure 3).

#### 3.2.3. Other Features

The axillary arch (Achselbogen of Langer) has been described in association with CEM (Table 1) on many occasions [3, 16]. The occurrence of this common aberration is in 7–13% of cases [5], where most specimens have demonstrated the presence of these anomalous muscles concurrently [3, 12, 18]. To date, only one specimen of bilateral CEM with no axillary arch muscle has been reported [5]. In our case, the specimen is unique being both bilateral and devoid of the axillary arch, while being associated with an anomalous fibrous band (tb) and untwisted PM.

### 3.3. Phylogeny

While the musculoskeletal system of humans has evolved over millennia to adopt bipedal terrestrial locomotion, the presence of anatomical anomalies may illustrate a missing link between junctions of evolution. Accordingly, we hypothesise that the presence of CEM associated with an abnormality of PM tendon insertion may demonstrate an important link in the evolution of terrestrial quadrupeds adapting to an arboreal lifestyle.

**Figure 4:**
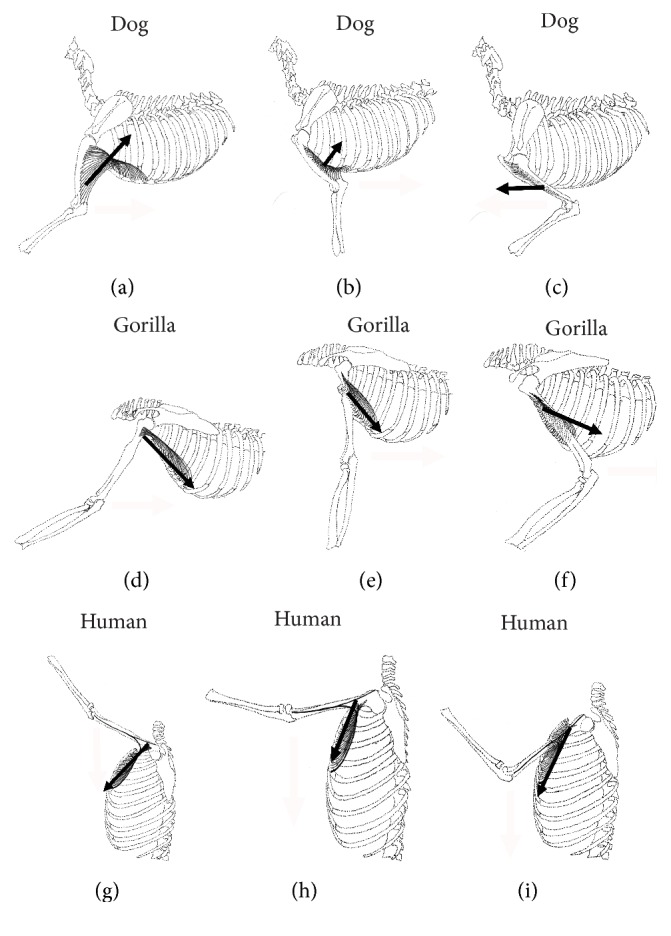
Collage of schematic drawings of the left thorax and upper limb of three different species. Black arrows depicting the direction of force of the pectoralis major muscle. At the end of a forelimb cycle of the dog (terrestrial quadruped), the forelimb needs to be drawn forward; hence the direction of PM is anterior. For the gorilla (arboreal bipedal), the forelimb can be further dragged down towards the midaxillary line due to the fiber direction of PM. In the human (terrestrial bipedal), one can draw the human's upper limb up to the midaxillary line well beyond the range of dog due to the change of insertion of the PM.

The pectoral muscle complex of lower mammals, which demonstrate a terrestrial quadruped locomotion pattern (Figure 4), has a broad insertion onto the humerus in comparison with primates who demonstrate an arboreal (Figure 4), relatively “erect” lifestyle. In lower mammals, the insertion of PM (in the form of pectoralis descendens and transversus) has been described to extend from the greater tuberosity to medial epicondyle of the humerus [12]. Furthermore, the insertion of PM has been shown to even extend down to the deep fascia of the forearm in some mammals (Figure 4) [15]. In humans, the sternocostal fibers of PM have migrated up under those fibers from clavicular and manubrial origins, resulting in the characteristic twist of the PM tendon [24]. However, in lower primates, the twisting of the PM tendon is seldom seen, which demonstrates the relationship to their quadruped ancestors [15, 25]. Miller and Oxnard both demonstrated that the pectoral muscles were directly attributed to the arboreal locomotion pattern seen in the primate kingdom in contrast to terrestrial quadrupeds. However, they did not specify the significance of the twist in the PM tendon contributing to the change in this more erect posturing [25, 26].

We hypothesise that the insertion of PM (which is the strongest muscle of the anterior chest) to the medial epicondyle of the forelimb would have synergised the drawing of the forelimb backwards (shoulder extension) on terrestrial quadruped motion (Figure 4). However, with the arboreal lifestyle and an erect posture adopted by the higher primates, the upper limbs were mainly used in climbing trees and grasping rather than walking. In an erect tree climbing motion, the forelimb requires drawing further towards the coronal plane of axis compared to walking on all fours. Therefore, having the PM inserted at the medial epicondyle would produce inefficiencies, during the final stages shoulder extension in an erect position (Figure 4). For example, during the final stages of upper limb drawing motion, the direction of muscle fibers would be against the direction of force that is required to extend the upper limb towards the coronal plane of the body. Conversely, if the PM was inserted higher on the humerus, it could be used in all the stages of climbing as depicted in Figure 4. Therefore, it is conceivable that the change in distal attachment of the PM tendon from the medial epicondyle to upper humerus associated with a twist correlates with adopting an arboreal climbing and erect posture from the terrestrial quadruped position. The change of the PM tendon has been demonstrated to be an actual twisting of its own caudal section under the cranial portion and not just a mere narrowing of its insertion [27].

In most CEM specimens (Table 1) with the untwisting of the PM tendon insertion, one other associated feature was the tuberoepicondylar band (described above) [3, 5, 12, 15, 16]. The presence of the CEM and the untwisting of the PM tendon have been explained to be two aspects of the same event by Landry Jr. [15]. We propose that the presence of this specific tb may indeed represent a vestigial tendinous fibrous band of PM insertion from the greater tuberosity to the medial epicondyle, thus demonstrating evolutionary change from lower quadrupeds [12, 25].

The presence of CEM, associated with the untwisting of PM along with the tuberoepicondylar band, highlights the possible evolutionary relationship humans have with lower primates and quadrupeds in terms of PM insertion. This supports the hypothesis and claim, put forward by Perrin, that CEM may represent the aberrant caudal fibers of PM [16], along with Chiba et al. who demonstrated that the nerve supply of CEM [3] is the same innervation with the lower fibers of PM [27]. The present specimen demonstrates the same pattern of innervation providing further support for the hypothesis that CEM is actually an atavistic remnant of the original PM seen in quadrupeds.

### 3.4. Clinical Significance

CEM has been reported on live subjects on a number of occasions: in two infants [13, 28]; a teenager [10]; a 31-year-old male weightlifter [11]; and a 30-year-old male [29]. Even though these patients presented with minimal symptoms, the clinical significance of such muscle, when present, has been claimed to be far more important in surgery, especially in breast surgery during axillary lymph node dissection [30]. Overall, the clinical significance of CEM is described in three main areas: muscle entrapment, neurovascular entrapment, and the significance of it on axillary surgery.

#### 3.4.1. Muscle Entrapment

Muscle entrapment due to the presence of CEM has been shown to restrict abductor movements at the shoulder joint during sports [29]. It is conceivable that CEM would limit shoulder abduction and cause tethering of the humerus as it spans across the axilla as a muscular band below the anterior axillary fold. Furthermore, CEM would reduce the range of movement of the axillary fascia when the arm is being abducted and may resist the flexion of biceps muscle as the tb crosses the long head of the biceps. Three cases reinforce such points, where a contracture of CEM in both a teenager [10] and two infants [13, 28] with limited shoulder mobility had symptomatic relief after surgical excision of the muscle.

#### 3.4.2. Neurovascular Entrapment

CEM has been documented to cause symptomatic nerve entrapment, where the tb was in direct contact with the ulnar nerve [11] and caused a tethering effect on the axillary neurovascular bundle. In the present specimen, the CEM was located away from the axillary neurovascular bundle (Figure 3) and was reinforced by a fascial sling. Moreover, in this specimen, having crossed the median nerve, brachial artery, and basilic vein, CEM did not tether the underlying nerves or blood vessels. Furthermore, it neither crossed nor was attached to the ulnar nerve; however it was closely related to the ulnar nerve anteriorly, but there were no fibrous connections between the two (Figure 2).

Although there are no reports of vascular entrapments due to this muscle, it may present as a practical obstacle during axilla surgery (i.e., treatment for cephalic arch stenosis) [8, 31]. Similarly, the presence of CEM may increase chances of lymphedema after mastectomy and axillary lymph node dissections due to its tendon crossing over the basilic vein.

#### 3.4.3. Axillary Surgery

Axillary lymph node excision is the standard surgical treatment for breast cancer with sentinel node macrometastasis [32]. Therefore, any anomaly that would pose a significant obstacle to this surgical procedure is noteworthy. CEM along with axillary arch and pectoralis quartus have been shown to be the main aberrant muscles that pose significant implications in axillary lymph node excision [30, 33]. The presence of an aberrant muscle in the axilla may be implicated in axillary surgery in two ways. First, an aberrant muscle spanning across the axilla may obstruct surgical access directly. Second, a muscle present at the edge of the surgical field may narrow the window of access [30]. Furthermore, as a cause of postoperative morbidity, the presence of CEM may cause lymphoedema, while the oblique band of CEM may compromise the venous drainage of the upper limb. Since postoperative lymphoedema is one of the most common postoperative complications following axillary surgery, CEM may be PM variation that surgeons should be cognisant of [2, 33].

## 4. Conclusion

We have provided a comprehensive anatomical description of the anatomy of the CEM in relation to its structure and neurovascular supply, while providing comparison and review of previous reports. Furthermore, we have argued its phylogenetic significance as a link between terrestrial and arboreal lifestyles in primate evolution, while the clinical relevance of this aberrant muscle was discussed in relation to shoulder mobility and neurovascular entrapment and as a potential aetiology of postsurgical lymphedema.

## Figures and Tables

**Table 1:** Summarized data of previous reported cases on chondroepitrochlearis muscle (CEM).

Author	Proximal attachments	Distal attachments	Untwist PM tendon	Axillary arch muscle	Tuberocondylar tendon	Blood supply	Nerve supply
Perrin [16]	7th rib, inferior fibers of PM, ext ob aponeurosis	Me	+	+	+		
Bryce [17]	6th costal cartilage	MIMS, Me		−	+		External anterior thoracic nerve
*Tobler (1902)*	Inferior fibers of PM	Me		+			Nerve to muscular arch
*Saar (1903)*	Inferior fibers of PM	Me					
*BÔSE (1904)*	Inferior fibers of PM	Me		+			
*Steinbach (1923)*	Abdominal fibers of PM	Me					
*Yokoh (1933)*	Inferior fibers of PM	Me		+			
*Tischendorf (1949)*	Abdominal fibers of PM	Me					Nerve to abdominal part of PM
Landry Jr. [15]	Costal cartilages	Me	+		+		
Aziz [18]	Inferior fibers of PM	Me	+	+	+		
Chiba et al. [3]	Inferior fibers of PM	Me	+	+	+		CBAP
Ohtani et al. [12]	Inferior fibers of PM	Me	+	+	+		CBAP
Voto and Weiner [13]	Costocartilages of ribs 7–10	Me			+		
Lin [10]	Inferior fibers of PM	Me		+	+		
Bergman [7]				+			
Spinner et al. [11]	6th and 7th ribs	Me		+	+		MPN, root of median nerve
Di Gennaro et al. (1998)	Inferior fibers of PM	Me			+		
Flaherty et al. [5]	5th costal cartilage	Me	+	−	+		MPN, root of median nerve
Nakajima et al. [9]	Inferior fibers of PM	Me		+			CBAP, intercostobrachial nerve
Sarikcioglu et al. [14]	5th and 6th costochondral junctions	Me	+	−		LTA	MPN
Loukas et al. [19]	lateral fibers of PM	Me				TAT	MPN
Samuel and Vollala [20]	Inferior fibers of PM	MIMS, Me	+	−		LTA	MPN
Barcia and Genovés [21]	5th costochondral junction	Me	−			LTA	MPN
Natsis et al. [6]	Costal cartilage at the lateral border of PM	Me					

PM: pectoralis major; ext ob: external oblique; Me: medial epicondyle; MIMS: medial intermuscular septum; LTA: lateral thoracic artery; TAT: thoracoacromial trunk; MPN: medial pectoral nerve; and CBAP: caudal branch of ansa pectoralis.

*Note*. *Data in italics* are extracted from Chiba et al. 1983 [3].
